# Mechanics of cell sheets: plectin as an integrator of cytoskeletal networks

**DOI:** 10.1098/rsob.240208

**Published:** 2025-01-29

**Authors:** Zuzana Outla, Magdalena Prechova, Katerina Korelova, Jakub Gemperle, Martin Gregor

**Affiliations:** ^1^ Laboratory of Integrative Biology, Institute of Molecular Genetics of the Czech Academy of Sciences, Prague, Czechia

**Keywords:** plectin, cytoskeletal crosstalk, epithelia, mechanobiology

## Abstract

Epithelia are multicellular sheets that form barriers defining the internal and external environments. The constant stresses acting at this interface require that epithelial sheets are mechanically robust and provide a selective barrier to the hostile exterior. These properties are mediated by cellular junctions which are physically linked with heavily crosslinked cytoskeletal networks. Such hardwiring is facilitated by plakins, a family of giant modular proteins which serve as ‘molecular bridges’ between different cytoskeletal filaments and multiprotein adhesion complexes. Dysfunction of cytoskeletal crosslinking compromises epithelial biomechanics and structural integrity. Subsequent loss of barrier function leads to disturbed tissue homeostasis and pathological consequences such as skin blistering or intestinal inflammation. In this article, we highlight the importance of the cytolinker protein plectin for the functional organization of epithelial cytoskeletal networks. In particular, we focus on the ability of plectin to act as an integrator of the epithelial cytoarchitecture that defines the biomechanics of the whole tissue. Finally, we also discuss the role of cytoskeletal crosslinking in emerging aspects of epithelial mechanobiology that are critical for the maintenance of epithelial homeostasis.

**Figure:**
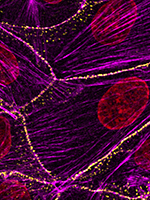

## 1. Introduction

Epithelia consist of layers of tightly connected cells that form a selective physical barrier essential for maintaining metazoan homeostasis. This broad category of tissues encompasses three distinct types: simple epithelia, stratified epithelia and pseudo-stratified epithelia. Simple epithelia, the most extensively studied, comprise a single layer of cells lining structures such as the intestine, body cavities and glandular tissues. Stratified epithelia, in contrast, consist of multiple cell layers and are exemplified by the epidermis. Pseudo-stratified epithelia, although composed of a single cell layer, appear stratified due to tightly packed cells of varying heights, as found in the upper respiratory tract.

Since epithelial sheets line the internal and external surfaces of the body and separate distinct compartments, epithelia are subject to considerable mechanical stress. Maintenance of epithelial barrier function therefore requires mechanical resilience. This relies on cytoskeletal networks, consisting of actin fibres, microtubules (MTs) and intermediate filaments (IFs) that define the mechanical properties and functional organization of epithelial cells. Mechanical robustness is further provided by cytoskeleton-associated cell junctions that seal intercellular spaces and interlink epithelial cells with the underlying basement membrane (BM). While apical tight junctions (TJs) and subjacent adherens junctions (AJs) are linked to actin filaments, desmosomes (DSMs), together with BM-associated hemidesmosomes (HDs), are connected to keratin IFs (KFs). The textbook view is that cell junctions together with cytoskeletal networks integrate epithelial sheets with BM into a structural and functional continuum.

Spatiotemporal architecture of cytoskeletal networks is controlled by cytoskeletal crosslinkers (so-called cytolinkers) of the plakin family [1]. Owing to their multimodular structure, these giant proteins (amassing up to 500 kDa) have capacity to bind all three cytoskeletal filaments and anchor the resulting networks to various cell structures (such as organelles and cell junctions). The best-studied plakin, a prototypical cytolinker plectin [2,3] (also known as IFAP300 [4] or HD1 [5]), was isolated as vimentin IF (VF)-binding protein from glia-derived cells in 1980 [6]. The name ‘plectin’ originates from the Greek word ‘πλεκτή’ (plectae), which means mesh or net [6]. Over the past decades, plectin has been implicated in many epithelia-affecting pathologies and multiple studies have highlighted the intimate relationship between plectin, cytoskeletal networks and epithelial physiology. Here, we review our current knowledge of plectin and how its structure and binding versatility are adapted to its mechanical and non-mechanical functions in epithelia. We also focus on molecular mechanisms underlying the major plectin-related, mechanical stress-driven pathological conditions. Finally, in the last sections of this review, we provide an overview of the emerging concepts in epithelial mechanobiology such as cytoskeletal crosstalk, adaptive reconfiguration of epithelial cytoarchitecture and mechanosignalling.

## 2. Plectin: a multifaceted crosslinker by design

**Figure 1.**
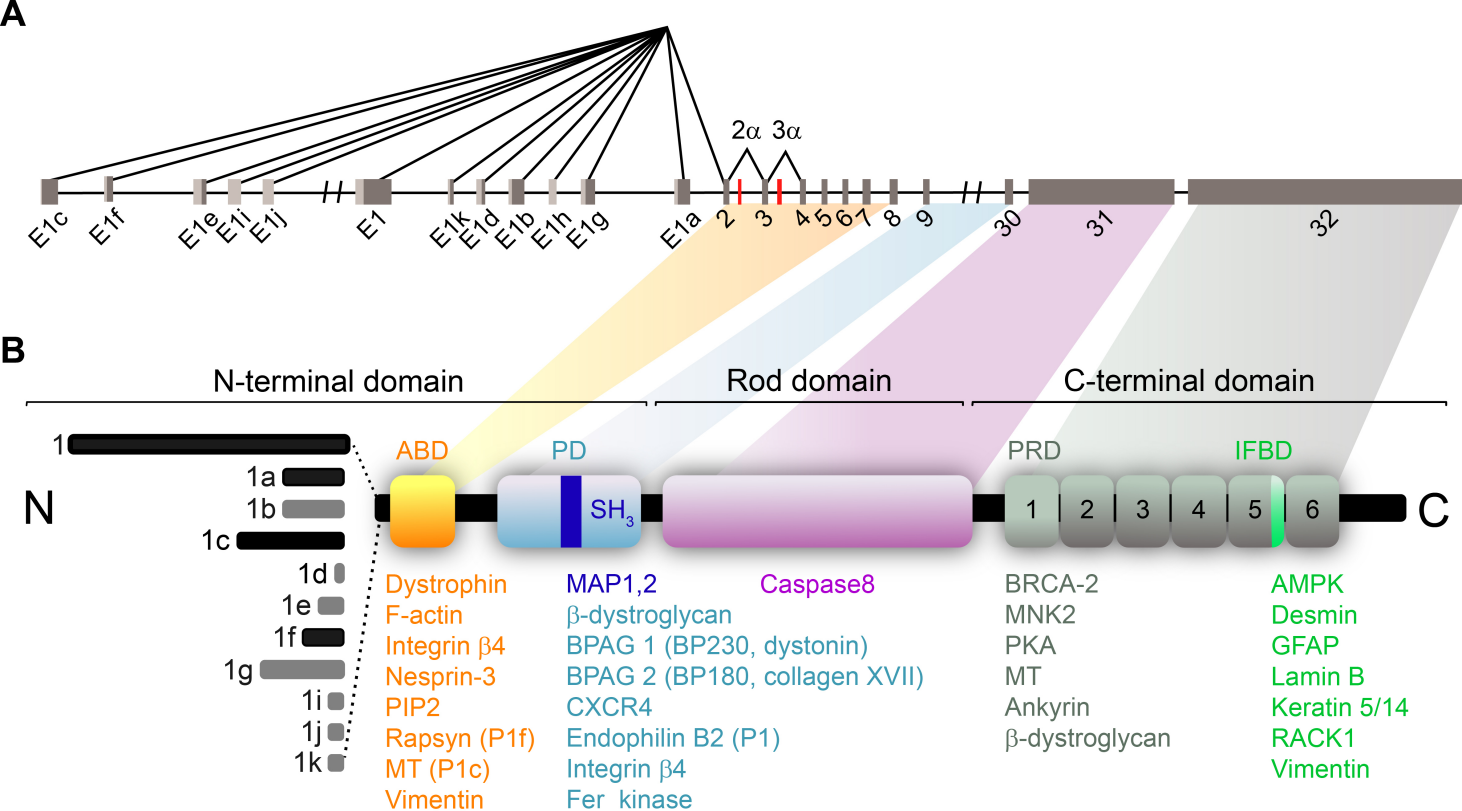
Schematic representation of plectin transcripts and encoded protein domains. (*A*) Schematics of the plectin transcripts. Twelve first exons spliced into exon 2 are shown. Untranslated regions (light grey) and two optionally spliced exons, 2a and 3a (red), are indicated. (*B*) Schematics of the plectin protein. The N-termini of isoforms predominantly expressed in epithelia are indicated (black). The N-terminal domain contains the ABD (yellow) and PD (light blue), which contains non-canonical SH3 domain (dark blue). Alternative splicing of exon 31 encoding the rod domain of plectin (pink) generates the rodless isoform. The C-terminal domain consists of six plectin repeat domains (PRD; grey), with the linker region of the fifth PRD containing an IFBD (green). The corresponding interacting partners are indicated below. The figure is not drawn to scale.

Plectin molecules adopt the general plakin modular structure with the N-terminal plakin domain (PD), the central coiled rod domain and the C-terminal globular domain (figure 1) (previously reviewed in [7,8]). These domains enable plectin to interact with all the major cytoskeletal components: actin fibres, IFs and MTs. The N-terminal segment contains the actin-binding domain (ABD) together with the canonical PD, which harbours the putative Src homology 3 (SH3) domain [9–11]. The IF-binding domain (IFBD) is located within a C-terminal region of six plakin repeat domains [12–16]. The additional VF-binding site is located within the ABD [17]. Although the IF-binding sites are well identified, the stochiometry of IF-plectin binding remains somewhat enigmatic [13,18]. The end of the C-terminal region contains the MT-binding Gly–Ser–Arg-containing repeats [19]. In addition, an isoform-specific MT-interacting site that overlaps with ABD in the N-terminal region has recently been identified [20]. The central rod domain mediates the dimerization of plectin molecules through coiled-coil interactions, leading to the lateral association with plectin oligomers [18,21]. However, in the context of the epithelial cell–cell junctions, our findings do not suggest plectin to form an anti-parallel dimer described previously in HDs [21,22].

In the early stages of apoptosis, the plectin rod domain is cleaved by caspase 8 [23]. Alternative splicing of exon 31 results in the rodless plectin variant [24]. All the plectin domains are multifunctional and interact with many proteins. For example, plectin recruits the cytoskeleton to cell junction complexes through association with integrin ß4 [25,26], bullous pemphigoid antigen (BPAG)1 and 2 [27–29], periplakin [30] or zonula occludens 1 (ZO-1) [31]. The interactions of plectin with other cytoskeleton-associated proteins, namely nesprin-3 [32], ankyrin [33], endophilin B2 [34,35], epithelial protein lost in neoplasm (EPLIN) [36] or MT-associated protein 2 (MAP2) [37] are also instrumental for the cytoskeletal configuration.

In addition to its organizational role, plectin is involved in signal transduction as a scaffolding protein, but also as a substrate of various serine/threonine protein kinases (such as Fer [38], AMPK [39], PKA and MNK2 [40]), or non-receptor tyrosine kinases (such as Src [41,42] and Pyk2 [41]). Furthermore, plectin can regulate kinase activity by sequestering its scaffolding proteins. For instance, plectin binding of RACK1 (the receptor and scaffolding protein of activated PKC) has been shown to regulate the PKCδ/Src/Erk2 pathway in keratinocytes [42,43]. For detailed information on plectin interacting partners see figure 1 and table 1.

**Table 1.** Summary of plectin interacting proteins according to identified interaction domain (see figure 1). CaM, calmodulin; SAXS, small-angle X-ray scattering; Co-IP, co-immunoprecipitation; GST, glutathione *S*-transferase; EM, electron microscopy; AchR, acetylcholine receptor; BMZ, basement membrane zone; FluoBACE, fluorescent protein-binding assay; LC-MS, liquid chromatography-mass spectrometry; IP, immunoprecipitation; MS, mass spectrometry; SILAC-MS, stable isotope labelling by amino acids in cell culture-mass spectrometry; EMSA, electrophoresis mobility shift assay; RBNS, RNA Bind-n-Seq; MALDI-MS, matrix-assisted laser desorption/ionization-mass spectrometry; DGC, dystrophin glycoprotein complex.

name	protein function	described function of interaction	method	references
***N-terminal region***				
calmodulin (P1a-specific)	calcium-binding messenger protein	integrin α6β4–plectin complex disruption F-actin–plectin binding inhibition	CaM-Sepharose pulldown, actin co-sedimentation assay, SAXS	[44,45]
**ABD**				
ß-dystroglycan	cell adhesion	desmin filament anchorage at sarcolemma	microtiter plate-binding assay	[46]
dystrophin (Utrophin)	cell adhesion	desmin filament anchorage at sarcolemma	Co-IP, GST pull-down	[46]
F-actin	actin cytoskeleton	F-actin network organization	actin-binding assay	[47]
integrin ß4	cell adhesion	HD assembly and stability, KF–HD linkage	crystallization, *in vitro* binding assays	[25,26]
nesprin-3	actin cytoskeleton	IF-outer nuclear membrane linkage	Co-IP, EM	[32]
PIP2	cell signalling	modulation of plectin–actin binding		[48]
rapsyn (P1f-specific)	AchR scaffolding protein	IF–AchR linkage, neuromuscular junction organization	Co-IP, GST pull-down, EM	[37]
MT (P1c-specific)	MT cytoskeleton	MT dynamics regulation	MT co-sedimentation assay	[20]
vimentin	IF cytoskeleton	vimentin network organization	affinity chromatography, crystallization	[17]
**plakin domain**				
BPAG 1 BP230- dystonin	cell adhesion, IF cytoskeleton	HD assembly	Co-IP	[27,29]
BPAG 2 BP180- collagen XVII	cell adhesion	BP180-β4 linkage support, epidermal BMZ organization	Protein–protein binding assay	[27,28]
CXCR4	chemokine receptor	CXCR4 trafficking and HIV-1 infection promotion	Co-IP, GST pull-down	[49]
endophilin B2 (P1-specific)	endosome maturation	perinuclear vimentin network organization, nuclear positioning	GST pull-down	[35]
integrin ß4	cell adhesion	stabilization of KF-HD linkage	yeast two-hybrid binding assay, Co-IP	[26,50]
MAP1, 2 (SH3 domain)	MT cytoskeleton	prevention of MT-MAPs binding	*in vitro* binding assays	[37,51]
Fer kinase	receptor Tyr kinase, proto-oncogene	negative regulation of Fer activity	Co-IP	[38]
**rod domain**				
caspase 8	apoptotic process	apoptosis-induced actin cytoskeleton reorganization	*in vitro* cleavage by recombinant caspases	[23]
**C-terminal domain**				
ß-dystroglycan	cell adhesion	desmin filament anchorage at sarcolemma	microtitre plate-binding assay	[46]
BRCA-2	DNA repair	prevention of micronuclei formation, centrosomal positioning	GST pull-down	[52]
MNK2	Ser/Thr protein kinase, MAPK signalling	plectin-IF binding modulation	yeast two-hybrid assay, CGP 57380 treatment	[40]
PKA	Ser/Thr protein kinase	plectin-IF binding modulation	yeast two-hybrid assay, H-89 and 8-Br-cAMP treatment	[40]
MT	MT cytoskeleton	MT destabilization	MT binding assay	[19]
ankyrin	actin cytoskeleton	costamere organization	yeast two-hybrid assay, GST pull-down	[33]
**IFBD**				
AMPK	Ser/Thr protein kinase, energy sensor	stabilization of AMPK γ1 regulatory subunit complex	yeast two-hybrid assay, Co-IP	[39]
desmin	IF cytoskeleton	desmin network organization	yeast two-hybrid assay, GST pull-down	[53]
GFAP	IF cytoskeleton	GFAP network organization	Co-IP, *in vitro* overlay assay	[13,54]
lamin B	IF cytoskeleton		solid-phase binding assay	[14]
K5/14	IF cytoskeleton	K5/14 network organization	yeast two- and three-hybrid assay, FluoBACE assays	[15,55]
RACK1	PKC regulation	PKC signalling regulation	yeast two-hybrid assay, Co-IP	[43]
vimentin	IF cytoskeleton	vimentin network organization	solid-phase binding assay	[13–15]
**unspecified**				
desmoplakin	cell adhesion, IF cytoskeleton	IF-DSM linkage	Co-IP, *in vitro* binding assay	[56]
dishevelled-2	Wnt signalling	dishevelled-2 stabilization	IP	[57]
Dlc1	GTPase-activating protein, tumor suppressor		HaloTag pulldown/LC-MS screen, IP	[58]
α-dystrobrevin (P1-specific)	IF cytoskeleton, DGC component	costamere organization	Co-IP, blot overlay assay	[59]
EPLIN	actin cytoskeleton	apical extrusion regulation	Co-IP	[60]
Fodrin	actin and MT cytoskeleton		Co-IP	[56]
FUS	DNA/RNA-binding protein	FUS localization and function regulation	GST pull-down	[61]
GPR56	cell adhesion		MS screen, Co-IP	[62]
K18	IF cytoskeleton	keratin network organization	Co-IP	[63]
K8	IF cytoskeleton	K8-mitochondria linkage, mitophagy promotion	Co-IP	[12]
KPNA2	nuclear importin subunit	lung metastatic potential promotion	SILAC-MS screen	[64]
MT (P1c-specific)	MT cytoskeleton	MT destabilization	MT co-sedimentation assay	[37]
NR3a	glutamate receptor subunit		yeast two-hybrid screen, GST pull-down	[65]
periplakin	cell adhesion, IF cytoskeleton	keratin network reorganization, cell migration promotion	Co-IP	[30]
Pyk2	non-receptor Tyr kinase	Pyk2 activation Src, actin ring formation and bone resorption	IP	[41]
RON-MST1R	receptor tyrosin kinase	disruption of plectin–integrin ß4 interaction, cell migration promotion	LC-MS screen, IP	[16]
Siah (P1-specific)	E3 ubiquitin-protein ligase		surface plasmon resonance analysis	[66]
α-spectrin	actin cytoskeleton		solid-phase binding assay	[51]
Src	non-receptor kinase, proto-oncogene	Src activation (plectin is in turn phosphorylated by activated Src)	LCMS screen, Halo-tag pull-down	[41,67]
SNRPA1	alternative splicing mediator	breast cancer promotion via Δexon 31 plectin	EMSA, RBNS	[68]
ß-synemin (P1-specific)	IF cytoskeleton	costamere organization	GST pull-down	[59]
ZO-1	cell adhesion, actin cytoskeleton		MALDI-MS screen	[31]

**Figure 2.**
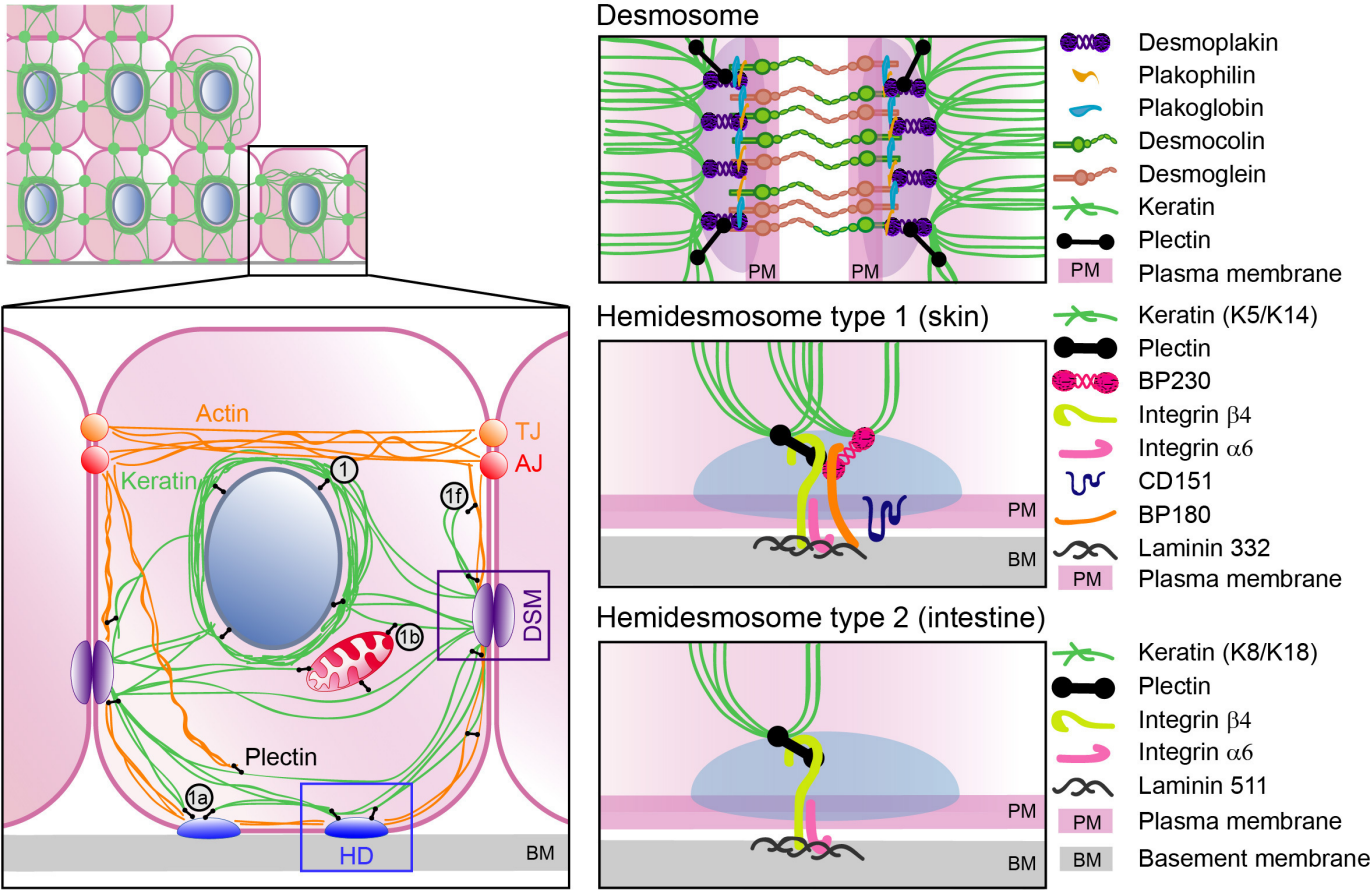
Schematic representation of plectin localization and plectin-mediated organization of cytoskeletal networks in epithelial cells. Plectin (black) crosslinks KFs (green) with the actin fibres (orange). Plectin also anchors cytoskeletal networks to cell junctions, such as HDs (blue; cell–ECM adhesion) and DSMs (purple; cell–cell adhesion), shown in more detail on the right. The localization of major plectin isoforms (black circles) is indicated. TJ, tight junction (orange); AJ, adherens junction (red).

The plectin locus [69] can generate at least 14 different protein isoforms (figure 1) by using alternative start sites and internal splicing. The alternative first exon sequences modulate the binding properties of subsequent ABD, thereby facilitating the targeting of individual plectin isoforms to diverse cellular structures [44,70,71]. In epithelia, the predominantly expressed isoforms are plectin 1 (P1), 1a (P1a), 1f (P1f) and 1c (P1c) [72]. Isoform P1 localizes to the perinuclear region [73] where it links IFs to the nuclear envelope via nesprin-3 and endophilin B2 binding [32,34,74]. Isoform P1a interacts with integrin ß4 within HDs—the complexes that mediate cell adhesion to the underlying dense extracellular matrix (ECM) organized into BM [26] (figure 2). In stratified epithelia, P1a is therefore predominantly found in the basal cell layer, where its staining signal co-aligns with the BM [73]. In an isoform-specific manner, P1a was found to restore the HD formation and attachment to the BM in plectin-deficient keratinocytes [73]. Another membrane-enriched isoform, P1f, tethers VF precursors to focal adhesions (FAs) in fibroblasts [71,75]. Such FA-associated KF assembly has also been documented in epithelial cells [76]. Remarkably, only re-expression of P1a and P1f isoforms restores the aberrant KF organization within the cell periphery in plectin-deficient epithelial monolayers [22]. In contrast to P1a, the P1c isoform is predominantly expressed in the suprabasal layers of stratified epithelia [73]. The P1c-specific exon, together with two additional exons 2α and 3α inserted into the ABD (figure 1), confers MT-binding ability to plectin [20]. P1c is involved in the regulation of MT stability, as its ablation in keratinocytes leads to higher MT resistance to nocodazole treatment and increased MT dynamics, thus affecting cell shape, division and growth [37]. For an overview of the isoform-specific plectin functions in different experimental models, see table 2.

**Table 2.** Summary of key features of plectin isoforms and their functions.

isoform uniprot	transcript name transcript ID	isoform-specific model	localization	phenotype	references
***P1*** Q15149 -1	*PLEC-201* ENST00000322810.8	P1 knockout mouse; myoblasts	perinuclear	perimyonuclear desmin network integrity, positioning and mobility of myonuclei, mechanotransduction	[34]
		P1 knockout mouse; dermal fibroblasts and T cells		cell migration	[77]
***P1a*** *Q15149-4*	*PLEC-202* *ENST00000345136.8*	P1a-re-expressing plectin knockout immortalized keratinocytes	HDs	HD-like protein complexes formation and stabilization	[73]
		EBS-Ogna knock-in mouse; epidermis	HDs	HDs formation and function, HD-KF linkage	[21]
		EBS-Ogna knock-in mouse; primary keratinocytes	HDs	HD-like protein complexes formation and stabilization	[21]
		P1a-re-expressing plectin knockout MDCK cells	cell–cell borders	circumferential keratin rim formation	[22]
***P1b*** *Q15149-5*	*PLEC-203* *ENST00000354589.7*	P1b knockout mouse; primary myoblasts and fibroblasts	mitochondria	mitochondrial morphology, mitochondrion-KF linkage,	[78]
		P1b knockout mouse; dorsal root ganglion neuron	mitochondria	mitochondrial subcellular positioning, mobility and morphology	[20]
		P1b knockout mouse; muscle fibres	mitochondria	mitochondrial fusion–fission machinery	[79]
***P1c*** *Q15149-2*	*PLEC-208* *ENST00000436759.6*	P1c-re-expressing plectin knockout immortalized primary keratinocytes	cell periphery, MTs		[73]
		P1c knockout mouse; dorsal root ganglion and hippocampal neurons	MTs	axonal MT dynamics regulation, neuritogenesis regulation, MT-mediated mitochondrial and vesicular transport, growth cone morphology; effect on long-term memory, cognitive functions and pain sensitivity of P1c knockout mice	[20]
		P1c knockout mouse; sciatic nerves	MTs	number and thickness of motor nerve fibres, motor nerve conduction velocity	[80]
***P1d*** *Q15149-7*	*PLEC-207* *ENST00000398774.6*	P1d knockout mouse; muscles	Z-discs	costamere alignment	[81]
		P1d knockout mouse; muscles		mitochondrial distribution, desmin networks arrangement	[79]
***P1f*** *Q15149-9*	*PLEC-205* *ENST00000356346.7*	P1f-re-expressing plectin knockout MDCK cells	cell–cell borders	circumferential keratin rim formation	[22]
		P1f-re-expressing plectin knockout fibroblasts	FAs	FA dynamics regulation, VF precursor anchorage	[75]
***P1k*** *A0A8I5KUE3*	*PLEC-215* *ENST00000693060.1*	P1k-expressing plectin knockdown SW480 colon carcinoma cells	podosome-like adhesions	F-actin assembly and podosome-like adhesion site formation	[82]

## 3. Keratin networks as a safeguards of epithelial integrity

The major component of the epithelial cytoskeleton are the KFs, which are believed to play a vital role in mechanical integrity at the cellular and tissue levels (previously reviewed in [83–85]). Keratins are encoded by 54 evolutionary conserved genes. Based on their sequence, keratins are divided into two distinct gene families (Type I and Type II). The tripartite structure of keratins consists of a conserved central α-helical rod domain flanked by the variable head and tail domains. The amphipathic properties of the central domain allow spontaneous heterodimerization of Type I and Type II keratins [86,87]. These highly stable heterodimers associate laterally into four non-polar antiparallel tetramers, which anneal longitudinally into filaments, which are further organized into bundles [88].

The structure of keratin provides keratin networks with unique physical properties. KFs are highly extensible and can be stretched almost three times without rupture [89]. Compared to MTs and F-actin, the shortest flexible length makes KFs the most flexible cytoskeletal polymers in epithelia [90,91]. KFs also undergo strain stiffening upon mechanical load to absorb and dissipate energy [91]. As evidenced by the deformation of epithelial sheets, KFs enable the re-stiffening of epithelial cells under tension, thus contributing significantly to epithelial elasticity and tensile strength [92].

The importance of KFs for tissue integrity is highlighted by the severe skin, liver and intestinal fragility that occurs in genetic diseases [93,94]. These are closely mimicked in many genetic mouse models. For example, mice lacking K5 or K14, the major keratin pair of the epidermis, suffer from skin blistering [95,96], resembling patients with epidermolysis bullosa simplex (EBS) [97,98]. The K8 deficiency/mutations in intestine of both mouse and human result in epithelial defects associated with inflammatory bowel disease [99–102]. In the liver, the mutation of K8 or K18 results in compromised hepatocyte integrity and increased susceptibility to injury [103–107], corresponding to K8/K18-associated human liver diseases [108–111].

## 4. Plectin governs epithelial keratin network architecture

**Figure 3.**
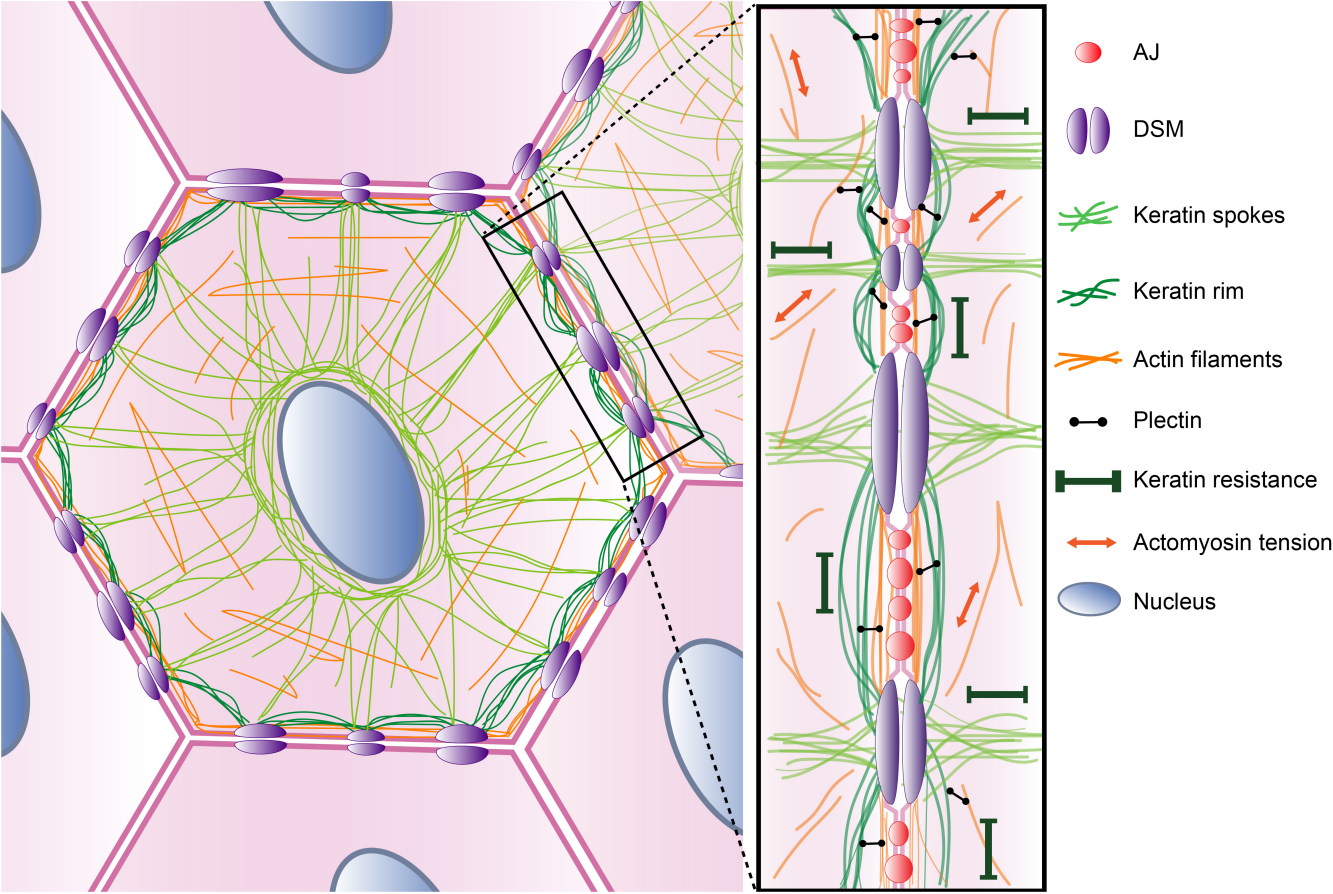
Schematic representation of the proposed model for plectin-mediated cytoskeletal organization and tensional homeostasis. In epithelial monolayers, the perinuclear KFs connect to the cell periphery via keratin radial spokes (light green) that are anchored to the membrane at DSMs (purple). Contractile actomyosin networks (orange) closely align with the plasma membrane. Plectin (black) interacts (via its N-terminus) with cortical F-actin and facilitates the formation of a circumferential keratin rim (dark green) that interconnects individual DSMs. Together, the interlinked web of cytoskeletal networks has the ability to dissipate local deformations, support the plasma membrane and protect the epithelial sheets from mechanical damage.

The unique mechanical properties are functionalized by plectin (and other plakins), which ensure the arrangement of KFs into highly organized networks. In epithelial cells, KFs are arranged in a so-called rim-and-spoke configuration in a plectin-dependent manner [22,112]. This consists of a subplasmalemmal circumferential keratin rim that associates with another subset of KFs that are aligned in dense radial spokes that span the cytoplasmic space between the nucleus and the peripheral compartments of the cell (figure 3) [112–114].

Our recent study [22] has shown that plectin-mediated crosslinking between keratin and actin peripheral networks is required for the formation of the circumferential keratin rim in epithelial monolayers. Furthermore, plectin stabilizes epithelial cytoarchitecture by anchoring KFs to cell adhesions (HDs and DSMs) and the nucleus [115], while condensing the keratin network via orthogonal crosslinking of individual filaments [42]. This is reflected in the collapse of the keratin network into more bundled and less flexible filaments upon plectin loss [22,42,116–119]. Overall, plectin inactivation results in circumferential keratin rim ablation, KF bundling, cell adhesion defects and general destabilization of aberrant keratin networks in epithelial cells [22,42,117,120,121].

Distinct populations of epithelial cells are characterized by unique keratin expression profiles. For instance, the basal layer of the epidermis expresses the keratin pair K5/K14, which is sequentially replaced by K1/K10 in the suprabasal layers [122,123]. Similarly, biliary epithelial cells (BECs) in the liver express five different types of keratins: K7, K8, K18, K19 and K23 [124,125]. Such redundancy prevents the assessment of the contribution of individual keratins to different cell/tissue properties (including mechanical properties), as suggested in the case of K10-, K19-, or K17-deficient mouse models [126–128].

The critical role of functional KF architecture is underscored by the phenotype of the liver-specific mouse model, in which plectin deletion was found to affect all keratin isotypes to the same extent [117]. In these mice, aberrant keratin networks in plectin-deficient BECs resulted in prominent biliary epithelial instability under cholestatic conditions. Such defects were not evident when only the K19 isotype was ablated [126]. Thus, plectin can be used as a unique tool to identify the role of keratins in complex systems. In contrast, other epithelial cells do not express such a broad spectrum of keratin isotypes. In these cases, the plectin-targeting phenotype mimics the keratin-related effects, as demonstrated by the destabilized keratin architecture of plectin-deficient hepatocytes expressing only the K8/K18 pair [117,129]. Similar changes have also been identified following plectin or keratin inactivation in the intestinal epithelium with K8, K18 and K19 expression [100,121,130].

## 5. Plectin in adhesion and cohesion of epithelial cells

The structural and functional integrity of epithelial sheets is secured by their coupling to the subjacent BM. Cell–ECM coupling is facilitated by HDs, multiprotein adhesion complexes that also play a crucial role in KF nucleation and anchorage [131,132]. In (pseudo)stratified epithelia, type I HDs are located in the basal cell layer and consist of integrin α6ß4, tetraspanin CD151 and the plakins BPAG1c (also known as BP230), BPAG2 (also known as BP180 or collagen XVII) and P1a [133]. In simple epithelia, type II HDs link the cells to the BM via the integrin α6ß4 and P1a only [134,135] (figure 2).

As a major component of HDs, plectin is highly expressed in all types of epithelia [136]. Therefore, the tissue-specific ablation of plectin in mice leads to the rearrangement of KFs, dramatic reduction of HDs and increased susceptibility to mechanical injury. As a result, compromised intestinal barrier [121], collapsed bile ducts and ductules [117], or detachment of the skin basal layer from the BM [73,137] have been found in plectin-deficient models. Plectin mechanically stabilizes epithelia not only through KF–HD coupling and KF configuration, but also by stabilizing HDs through self-association of its rod domain into more stable oligomers [21].

Functional epithelial barriers and tissue homeostasis also require robust sealing of the intercellular spaces. This is achieved by a joint effort of cytoskeleton-bound cell–cell junctions: F-actin-linked TJs and AJs and KF-linked DSMs, distributed along the border of neighbouring epithelial cells (figure 2). Apically located TJs consist of transmembrane junctional adhesion molecules, occludins and claudins, which are linked to the actin cytoskeleton by ZO proteins [138–141]. The cohesion of AJs is ensured by the trans-dimerization of E-cadherin transmembrane proteins that bind to p120-catenin or ß-catenin. The F-actin–AJ linkage is facilitated by α-catenin [142]. DSMs comprise the transmembrane adhesion proteins desmogleins and desmocollins, the DSM plaque of plakoglobin and plakophilin proteins and the KF-linked desmoplakin [143–145] (figure 2).

Plectin has been shown to associate with DSMs and to form complex with desmoplakin and other junctional factors [4,56,146,147]. Multiple studies have recently converged on identifying the indispensable role of plectin in intercellular adhesion function and cell–cell cohesion [22,117,121,148]. In mouse endothelium, the plectin depletion leads to AJ and TJ distortion paralleled by increased vascular permeability [148]. Impaired epithelial barrier function and dilation of intercellular spaces have also been described in mouse plectin-deficient biliary and intestinal epithelia, where defects in TJs, AJs and DSMs have been noted. Therefore, dysfunctional cell cohesion, together with aberrant KF architecture and weakened cell–ECM adhesion, favours epithelial fragility, that manifests in pathologies such as ulcerative colitis or cholestasis [117,121]. Furthermore, less effective upregulation of desmoplakin in a plectin-deficient model of liver injury, suggests a role for plectin in maintaining DSM homeostasis [117]. As shown in Madin-Darby Canine Kidney (MDCK) cells, ablation of plectin leads to increased tensile loading on DSMs and compromises the mechanical integrity of epithelial sheets [22].

It is well-recognized that plectin exerts numerous non-mechanical functions which are essential for the maintaining of epithelial homeostasis. For instance, plectin may influence TJ stability and epithelial barrier properties by acting as a regulatory scaffold for AMPK regulatory subunit γ [39,149].

## 6. Clinical manifestation of plectin mutations

More than 100 mapped mutations in the human plectin gene (*PLEC*) result in plectinopathies [150,151], a group of complex multisystem disorders that primarily affect tissues exposed to mechanical stress. The most common plectinopathy is EBS, which manifests clinically with severe epidermal blistering of early onset. The autosomal recessive variant of EBS is associated with muscular dystrophy (EBS-MD), myasthenic syndrome (EBS-MD-MyS), pyloric atresia (EBS-PA) or congenital myasthenia (EBS-CMS). These plectinopathies are often caused by mutations in exon 31, which encodes the rod domain, leading to nonsense-mediated mRNA decay (reviewed in [152,153]). In contrast, the autosomal dominant EBS-Ogna disease is caused by a site-specific missense plectin mutation in exon 31 and manifests exclusively as skin fragility [152,154]. Progressive familial intrahepatic cholestasis has also been reported [155,156].

Only five mutations have been so far identified within the alternative first exon of a specific plectin isoform [151]. For example, homozygous deletion of P1f causes an autosomal recessive limb-girdle muscular dystrophy (LGMD) associated with progressive muscle weakness without any dermatological component [157]. A similar phenotype was reported for another P1f-specific mutation (c.58G>T, p.E20X) where patients suffered from LGMD and respiratory symptoms [158]. Several other *PLEC* mutations have been linked to cardiomyopathy and malignant arrhythmias [151,159]. The homozygous nonsense mutation in P1a (c.46C>T, p.Arg16X) causes autosomal recessive skin-only EBS with no effect on mucous membranes, heart and muscles [160]. Recently, novel missense mutations in P1-specific N-terminal sequence, exon 31 and exon 32 were identified in patients suffering from hearing loss, without any additional clinical conditions [161].

## 7. Mouse models of plectinopathies

In recent years, several different mouse models of plectin deficiency have been generated and have been instrumental in our efforts to investigate the role of plectin in tissue and cell mechanobiology. Mice deficient in all plectin isoforms (plectin-null) die early in postnatal development due to internal blistering of the oral cavity that prevents food intake [137,162]. At the cellular level, plectin-null mice exhibit rupture of basal keratinocytes, reduced number of HDs and impaired stability of the keratin networks. In addition, plectin-null mice exhibit structural abnormalities in the heart and skeletal muscle [162]. The severity of the phenotype prevents any in-depth analysis, and therefore this model is not useful for studying human diseases caused by mutations in the plectin gene.

To circumvent the neonatal lethality of plectin-null mice, four types of isoform-specific knockout mice, lacking either P1, P1d, P1c or P1b, have been generated [77,78,80] (reviewed in [163]), significantly broadening the basis for dissecting the isoform-specific roles of plectin in epithelia. A plectin knock-in mouse model has been established to mimic the EBS-Ogna disease [21]. The EBS-Ogna mutation increases the susceptibility of the P1a rod domain to proteolytic cleavage in the epidermis, resulting in reduced P1a protein levels in basal keratinocytes. Furthermore, the mice expressing a rodless variant of plectin, which has been identified in a subset of EBS-MD patients with milder prognosis [164], develop without signs of skin blistering or HD changes. This finding suggests a compensatory effect of rodless plectin in the setting of full-length plectin deficiency [165]. Taken together, analyses of these models suggest that sufficient levels of plectin expression, as well as its interaction with HDs in keratinocytes, are critical for maintaining epidermal integrity.

Apart from isoform-specific and knock-in mouse models several conditional tissue-specific models have been used in complex studies of the role of plectin in epithelia. For example, models of both constitutive and tamoxifen-induced plectin ablation in intestinal epithelial cells exhibit detachment of the epithelial sheet from the BM, loss of intestinal barrier integrity and subsequent spontaneous development of a colitic phenotype [121]. It is therefore not surprising that the plectin expression levels were found to be negatively correlated with the severity of ulcerative colitis in human patients [121]. Liver-specific plectin knockout mice revealed disruption of apicobasal KF polarity and compromised cell–cell junctions in BECs, accompanied by the collapse of the ductular lumen and misshapen bile canaliculi [117]. When challenged in a model of obstructive cholestasis, plectin ablation exacerbated biliary damage and resulted in markedly dilated bile ducts with more frequent ruptures and increased number of biliary infarcts in a bile duct ligation model. This model resembles the condition of patients who carry the compound mutation in the plectin SH3 domain and rod domain and suffer from intrahepatic cholestasis [155,156].

## 8. Novel perspectives on epithelial mechanobiology

Over the past few decades, we have begun to understand the molecular basis of the high mechanical resistance of epithelial tissues. We now recognize that this is primarily achieved through the cytoskeletal networks and cell junctions, which are physically and functionally integrated by cytolinker proteins. In the following paragraphs, we discuss the emerging concepts of epithelial biomechanics, including the significance of the interplay among distinct cytoskeletal networks, the role of cytoskeletal crosslinking in maintenance of tensional homeostasis and the adaptive response of epithelia to mechanical stress. Finally, as increasing evidence suggests that plectin can regulate signalling pathways in response to mechanical cues, we propose a concept in which plectin represents a new class of versatile mechanosensors that allow the control of tensional homeostasis required for epithelial integrity.

### 8.1. Interplay between epithelial actin and keratin cytoskeletal networks

Previous attempts to define the role of cytoskeletal networks in the mechanical homeostasis of epithelial sheets have mainly been limited to their individual components (i.e. actin fibres, KFs and MTs) and have therefore provided only a fragmentary insight into the complexity of epithelial mechanics. In recent years, there has been increasing evidence that key features of complex networks arise from the cooperativity of structurally and functionally distinct cytoskeletal subsets that are physically integrated by cytoskeletal crosslinkers [7,22,136,166,167]. Such an interconnectedness is currently proposed for the plectin-mediated crosstalk between F-actin and IFs in motile vimentin-expressing cells, where the VFs provide a load-bearing ‘meshwork’ that supports the contractile actomyosin system [168–172]. This raises the question of whether a similar functional coupling exists between epithelial keratin and actin networks.

In polarized epithelial cells, KFs and F-actin are tightly intertwined within the circumferential cytoskeletal network [22,173]. The subplasmalemmal space is dominated by a prominent actin belt, that is closely associated with mature AJs [174,175] (figure 2). The cortical actin belt (so-called actin cortex) is composed of thick bundles of F-actin that are associated with non-muscle myosin II filaments and running parallel to the cell membrane [174]. This organization of the actin cytoskeleton enables force generation and controls the integrity of intercellular junctions [176–178]. Aligned parallel to cortical F-actin, the subset of interdesmosomal KFs assembles into a keratin circumferential rim [22,112], thus forming a mechanically robust KF–DSM network. We found that plectin decorates F-actin structures and colocalizes with circumferential keratin in MDCK cell monolayers [22]. Recently, a similar organization has been described also in bladder umbrella cells [179]. Based on these findings, we have proposed a model [3,22] in which plectin, by virtue of its structure, interacts simultaneously with KFs (via C-terminal IFBD) and cortical F-actin (via N-terminal ABD), thus securing close apposition of both circumferential structures (figure 3).

In the proposed model, plectin integrates keratin and actin subplasmalemmal networks to provide mechanical support for the plasma membrane and cell–cell contacts. Such a hardwired circumferential network has been reported in many epithelial sheet-forming cell types, including keratinocytes [114], intestinal epithelial cells [180], pancreatic exocrine acinar cells [181], bladder umrella cells [179] or hepatocytes [182,183] (for review, see [112]). The structural dependence of the keratin circumferential rim on the actin belt became apparent upon the removal of plectin-mediated interlinkage. Hence, both genetic (CRISPR/Cas9 system) and pharmacological (plecstatin-1 [184]) inactivation of plectin led to complete disruption of subplasmalemmal keratin structures [22] in epithelial monolayers grown from MDCK cells, BECs and mammary epithelial cells. Disruption of the actin belt by low doses of the actin-depolymerizing drug latrunculin A had a similar effect [22]. As the keratin circumferential rim is closely associated with DSMs, ablation of interdesmosomal KFs resulted in their uneven patterning. Furthermore, DSM-anchored radial keratin spokes collapsed and laterally aligned into thick keratin bundles, as previously observed in various plectin-deficient cell types [42,116–119]. Together, these observations identified the keratin circumferential rim as a cortical actin belt-dependent framework responsible for the spatial organization of KF–DSM networks.

The physical engagement between peripheral actin and keratin networks has important consequences for cytoskeleton-linked cell–cell junctions, which in turn affect cytoskeletal networks. For instance, it has recently been shown that KFs coupled to DSMs and the actin cytoskeleton linked to AJs cooperate to enable efficient mechanotransduction in epithelia, where DSM-anchored desmoplakin is required to activate RhoA at AJs in a response to tensile stimulation [185]. This is consistent with the previously reported increase in actomyosin contractility in cells where DSM–KF linkage was strengthened, while disruption of DSM–KF interaction resulted in reduced cellular forces [186]. Since keratin network itself is required for mechanosignalling [118] and a loss of several DSM components, such as desmoglein 1 [187], desmoglein 2 [188], desmoplakin [189,190] or plakophilins [191,192] have been associated with a perturbed actomyosin network, it is evident that the interconnection of KFs and DSMs is important for the organization of the actin cytoskeleton in epithelia.

The state of the actomyosin network simultaneously affects the DSM–KF networks. Partial inhibition of actomyosin contractility in keratinocytes and MDCK cells has been shown to reduce DSM protein turnover, affecting DSM formation and growth [193]. Meanwhile, increased actomyosin contractility, caused by the absence of aPKCλ, resulted in the reorganization and reinforcement of KFs and increased mechanical resilience in stratified epithelia [194]. Notably, increased actomyosin contractility conveyed by subplasmalemmal networks to junctions has been proposed as one of the key mechanisms driving epithelial barrier disruption during various pathophysiological conditions, such as mucosal inflammation [22,195,196].

Functional cooperativity of peripheral cytoskeletal networks is particularly required in cellular processes that load membranes with internally generated mechanical stress, such as phagocytosis or cytokinesis. Recently, K8 has been reported to interact with F-actin via plectin, to mechanically support the actin fibres and enable successful autophagosome–lysosome fusion [197]. Strikingly, VFs were also shown to form a plectin-dependent subcortical layer to control the organization of the actin cortex and the mechanics of the cortex during mitosis [198]. This suggests that the overall concept presented here is not limited to KFs and epithelia. Thus, plectin-mediated crosslinking of peripheral networks is expected to play a central role in cellular events that require increased mechanical support of the plasma membrane (such as invagination, immune response, cell division, or synapse development).

Although the first lines of evidence suggest the cooperativity of the subplasmalemmal KF and actin networks in regulating epithelial mechanics, direct evidence that physical coupling with KFs determines properties of the actomyosin network at the nanoscale level is currently lacking. This is a surprising gap in the knowledge, as both *in vitro* and theoretical studies suggest that actin network architecture and crosslinking levels are key regulators of contractile tension generation (for review, see [199]). While minimal crosslinking is required for contraction, excessive crosslinking limits contractile tension generation [200]. Therefore, the same actin crosslinker (such as α-actinin, fascin, filamin or fimbrin [201]) can either promote or inhibit the contractility of the actomyosin network [202]. Spatiotemporal changes in ‘network connectivity’ [202] facilitate the switching between contraction modes (sarcomeric versus buckling mechanisms [199]) and determine the length scale of contractions with the magnitude of the developed stresses [199]. Further studies are needed to investigate whether plectin-mediated integration of subplasmalemmal networks can play an analogous role, where local stabilization of F-actin by plectin would allow local modulation of contractility and tension.

### 8.2. Cytoskeletal crosslinking and tensional homeostasis

Plectin-mediated crosslinking integrates cortical keratin and actin networks to orchestrate the formation of epithelial keratin into a rim-and-spoke configuration [22,112]. The coupling of mechanically distinct polymers allows a dynamic balance between the contractile forces generated by actomyosin and the mechanical resistance provided by the KFs and is a physical prerequisite for the generation of an isometric tension within the network [203] (figure 3). The crosslinker hardwiring thus facilitates the establishment of a tensional homeostasis that stabilizes cell structures and maintains cell integrity [203–205].

As the actin and keratin networks of neighbouring cells within the epithelial sheet are interconnected via intercellular junctions, intrinsically generated forces can propagate on a multicellular scale to generate tissue-scale tensions [206,207]. Tissue can be considered a so-called ‘tensegrity structure’ (from tensional integrity [203]), where tensional homeostasis governs the structure of living systems [203–205]. Not surprisingly, several studies have identified plectin-controlled cortical networks as central to tension distribution and maintenance of tensional homeostasis at both the cellular and tissue levels (for review, see [207,208]). Exposure of epithelial sheets to externally generated forces (for instance, during physical trauma, wound healing or epithelial morphogenesis) places high demands on the stability of well-formed (i.e., properly crosslinked) cytoskeletal networks and cytoskeleton-linked intercellular junctions.

Experimental techniques for measuring tension in epithelial sheets have expanded dramatically in recent years. Among these, the laser ablation technique has proven to be a very convenient method to study epithelial mechanics without direct contact with the sample. A laser beam is used to cut subcellular, cellular or supracellular structures (including contractile ring [209], intercellular adhesions [210], ventral stress fibres [211] or cell cortex [212]), where the dynamics of the recoil of the ablated structure is indicative of its tensile properties [213]. Traction force microscopy [214] can be used to determine the force patterns exerted by individual cells, cell colonies as well as monolayers of epithelial cells. From the force balance between cell–substrate and cell–cell interactions, tensile stress at cell–cell borders can be calculated using monolayer force microscopy [215–220]. In an alternative approach, Förster resonance energy transfer (FRET)-based tension sensors [221] (mostly derived from cell junction constituents) can be used to quantify tension across intercellular junctions. Such measurements rely on the tension-induced unfolding of the inserted tension module and can provide a single-molecule, pN-scale readouts [222]). Currently, the growing number of junctional tension sensors includes vinculin [221], talin [223], E-cadherin [224], desmoplakin [225], desmoglein [226] or ZO-1 [227]. This rapidly expanding toolbox provides an opportunity for combinatorial experiments, allowing researchers to measure and compare forces at different cell junction types (such as AJs and DSMs [22]), providing a more detailed insight into the distribution of tension within epithelial sheets.

Only little is known about the forces acting across intercellular junctions in epithelial sheets. However, intrinsic tension at junctions arises when adhesions are coupled to the contractile cytoskeleton. For instance, using the E-cadherin FRET tension sensor, Nicolas Borghi *et al.* [224] demonstrated that the actomyosin cytoskeleton constitutively exerts pN-scale tension on individual E-cadherin molecules at the plasma membrane. When exposed to externally applied stretch, E-cadherin tension increased specifically at intercellular junctions, supporting the hypothesis that AJs transmit intracellular as well as extracellular forces. An increase in the tensional loading on E-cadherin was later described not only upon externally applied stretch but also in between epithelial cells within spheroids, spontaneously formed 3D structures with a fluid-filled central lumen [228]. In contrast to actin-associated AJs [207,224], KF-linked DSMs appear to experience only little (desmoglein FRET sensor [226]) or no (desmoplakin FRET sensor, [225]) strain under steady-state conditions. Under externally applied mechanical stretch, DSMs experience significant tensile stress [225], supporting the widely accepted view that the KF–DSM network is designed to absorb tensile stress and protect the mechanical integrity of epithelial sheets.

Disruption of tensional homeostasis often acts as a major trigger/driver of the biomechanical traits of numerous diseases. Probably the best-documented pathology in which dysregulation of biomechanical properties is associated with disease onset and progression is cancer [229]. Cortical tension influences several aspects of cancer progression, including cancer cell extrusion [230,231], epithelial–mesenchymal transition [232] and cell migration plasticity [233]. Furthermore, tight control of the biomechanics of the cell cortex is required for proper cell division [198,234,235]. Further studies have implicated dysregulation of cortical contractility in developmental defects [236] or immunodeficiency [237]. Unsurprisingly, epithelial instability, which is closely associated with several plectinopathic disorders (including skin blistering, obstructive cholestasis and ulcerative colitis [150]) arises from aberrant cytoskeletal architecture in which tension imbalance may be perceived as causative rather than consequential.

In previous sections of this review, we described that the loss of the circumferential keratin rim upon plectin inactivation is associated with the prominent bundling of KFs, leading to a reduced number of radial spokes. Interestingly, this pathological reconfiguration of KFs can be alleviated by experimentally reducing actomyosin contractility by blocking myosin II or inhibiting upstream Rho-associated kinase [22]. This suggests that tensile stress rather than the loss of orthogonal filament crosslinking [42] drives the collapse of keratin networks in epithelia with disabled plectin. Furthermore, using the E-cadherin and desmoplakin FRET sensors, we have shown that collapsed keratin spokes with actin fibres transmit increased and unevenly distributed cytoskeletal tension to DSMs and AJs [22]. This then leads to a profound perturbation of cell/tissue mechanics and prevents long-range force propagation [238], which is required for coordinated cytoskeletal stress adaptation [204]. As apparently tensed KFs have also been found in ß4 integrin-inactivated keratinocytes [119], loss of tensional equilibrium may also account for other cytoskeletal defects such as the keratin ‘fragile network’ [239,240] and the ‘sparse network’ [241], which were previously linked to skin fragility in EBS patients.

### 8.3. Adaptive response to mechanical stress

Epithelial sheets are designed to withstand substantial, dynamic external forces in the form of tensile, compressive and shear stresses. These can be tensile and compressive forces during morphogenesis or organ growth, mechanical friction that affects the epidermis, or shear stresses within internal organs (previously reviewed in [207,242]). Unlike cancer cells, where mechanical stress often leads to nuclear ruptures associated with DNA damage [243,244], epithelial monolayers can withstand extreme mechanical stresses that lead to large deformations without signs of damage [92,245]. This is achieved thanks to an adaptive process, which consists of several layers of protective responses including supracellular alignment, cytoskeletal rearrangement, reinforcement and nuclear adaptation (mainly chromatin reorganization) [246]. Each level of this adaptive response requires coordinated cytoskeletal regulation and the interplay of well-formed cytoskeletal networks.

When exposed to sustained uniaxial stretching, confluent epithelial sheets undergo supracellular monolayer reorganization, with cells rearranging their longer axes perpendicular to the direction of stretch [246,247]. Such reorganization is also associated with a gradual, time-dependent reorientation of F-actin and nuclei perpendicular to the direction of stretch, and reorientation of AJs at an angle of 45° to the stretch axis [246]. Single cells exposed to two-dimensional stretching also reorient their body axes perpendicular to the applied stretch [248,249]. This reorientation is both preceded and dependent on cytoskeletal reorientation and reinforcement [250]. Interestingly, all three cytoskeletal networks reorient in response to cyclic stretching with different dynamics. Actin filaments, as the most dynamic cytoskeletal network, reorient first, followed by MTs and IFs reorient last [250,251]. When comparing the extent of cytoskeletal reorganization between individual networks, cyclic stretching most affected the actin cytoskeleton, much less MTs and VFs were mostly only affected by whole-cell reorientation [252]. Surprisingly, while MTs do reorient in response to cyclic stretch, the cell reorientation does not depend on them [253,254], but rather relies on the integrity of the actomyosin network [253,255]. Such reorganization and alignment of cytoskeletal networks are thought to minimize passively stored elastic energy, thereby reducing mechanical strain on the cytoskeleton and preventing mechanical damage [256].

The strain-induced spatial reorganization of cytoskeletal networks is complemented by a biochemical response. Cell stiffening and increased mechanical resilience are largely regulated by the polymerization/depolymerization rate of actin fibres, the degree of cytoskeletal crosslinking (both between filaments of the same type and between filaments of different cytoskeletal networks), or changes in the expression level of cytoskeletal components [257]. Mechanical stress, including cyclic stretching, activates the RhoA signalling pathway, leading to the assembly of actin stress fibres and reinforcement of the actomyosin cytoskeleton [177,246,258–260]. Remarkably, K18 and the keratin-associated Rho GEF Solo have been shown to be required for efficient force-induced activation of RhoA [261], demonstrating the interdependence of cytoskeletal networks for efficient adaptation to mechanical stress. Strain-induced activation of RhoA signalling associated with the increased cytoskeletal tension and the actin polymerization, then initiates mechanosensitive transcription through the YAP/TEAD, AP-1 and MRTF/SRF pathways [258,262,263]. While MRTF (transcriptional coactivators of SRF transcription factor) activation requires low levels of G-actin, as their interaction with G-actin retains these coactivators in the cytoplasm [264,265] (reviewed in [266]), nuclear accumulation of YAP is primarily regulated by increased actomyosin contractility [267,268]. Since the target genes of the YAP/TEAD and MRTF/SRF pathways are mostly cytoskeletal components, the activation of such tension-sensitive transcriptional programmes results in the reinforcement of cytoskeletal networks and a long-term adaptation to mechanical stress.

Previous studies on stretch-induced cytoskeletal rearrangement have mainly focused on actin stress fibres [241,250,260], while the adaptation of KFs [240,250,251] has received only little attention. Both increased expression of KF–DSM network components and their reorganization have been described. Remarkably, the stretch-induced expression of KF–DSMs network components is dependent on the actomyosin cytoskeleton as several proteins of KF–DSM network (including keratin, desmoplakin, desmoglein and plakophilin) are regulated by mechanosensitive transcription pathways, activated by increased actomyosin tension [269–272]. Mechanical stress also induces the reorganization of the KF–DSM network. In keratinocytes, cyclic stretching has been shown to cause the thickening and compaction of keratin spokes [240]. Similarly, in a subset of extremely stretched MDCK cells, KFs form unusually straight and thick spokes that span between the perinuclear region and the plasma membrane [92]. Furthermore, MDCK monolayers in response to stretch substantially enrich KFs at the plasma membrane, where they co-align with DSMs [22]. Subplasmalemmal KF enrichment was accompanied by a redistribution of the cytoplasmic pool of plectin towards the circumferential cytoskeleton at cell–cell borders. Taken together, these data suggest that upon stretch, the more abundant plectin integrates newly recruited KFs with the pre-existing circumferential rim and cortical F-actin to reinforce the peripheral cytoskeleton and mechanically challenged intercellular junctions. These experiments also provide the first evidence that plectin-mediated crosslinking of actin and keratin networks is essential for strain-induced cytoskeletal reorganization and reinforcement of cell cohesion, both of which are required for effective epithelial mechanoprotection.

The interconnected network of three distinct cytoskeletal systems, each with unique viscoelastic properties, forms a complex network that must respond to mechanical stress in a coordinated manner. All three cytoskeletal networks contribute to tissue resilience, even under low levels of mechanical stress. However, it is the network of IFs, in particular KFs and lamins, that is responsible for resilience under higher stresses, as both actin filaments and MTs disassemble under strain [273,274]. Compared to F-actin and MTs, IFs are much more flexible due to the shorter persistence length of the filaments [275,276]. Keratins are also highly extensible and can be stretched up to three times before breaking [277]. In addition, IFs stiffen under strain [278,279], which is a key factor in mechanoprotective adaptation. To prevent mechanical damage, the hyperelastic and resilient KFs need to be interconnected with cytoskeletal components that can rapidly recover and restructure (such as the actin cytoskeleton). Such an interconnected network can dissipate local deformation and slow down the viscoelastic relaxation to protect organelles, including nuclei that encapsulate the genetic material, from mechanical damage, such as membrane rupture or DNA damage. Overall, the multiple layers of adaptive responses to mechanical stress, which include supracellular patterning, cytoskeletal reorganization and stiffening, as well as changes in gene expression and nuclear adaptation, require a coordinated response from the interconnected cytoskeletal networks to effectively protect epithelia from mechanical damage.

### 8.4. Plectin as a mechanosensor

The adaptive response of epithelia to mechanical stress requires epithelial cells to sense a wide range of both extrinsic and intrinsic forces. These mechanical stimuli typically elicit mechanotransduction responses through stretch-sensitive channels or junction-associated proteins that act as mechanosensors. Mechanosensing involves the ability to translate physical stimuli into biochemical signalling via force-induced modifications or conformational changes (for a general review, see [280,281]). The capacity of plectin to transmit tension and serve as a scaffolding platform for a multitude of signalling molecules, together with its location within mechanosensitive hubs (such as FAs and HDs), makes plectin an ideal candidate for a highly versatile mechanosensor. Furthermore, the mechanosensitive versatility of plectin could be further extended in an isoform-dependent manner.

**Figure 4.**
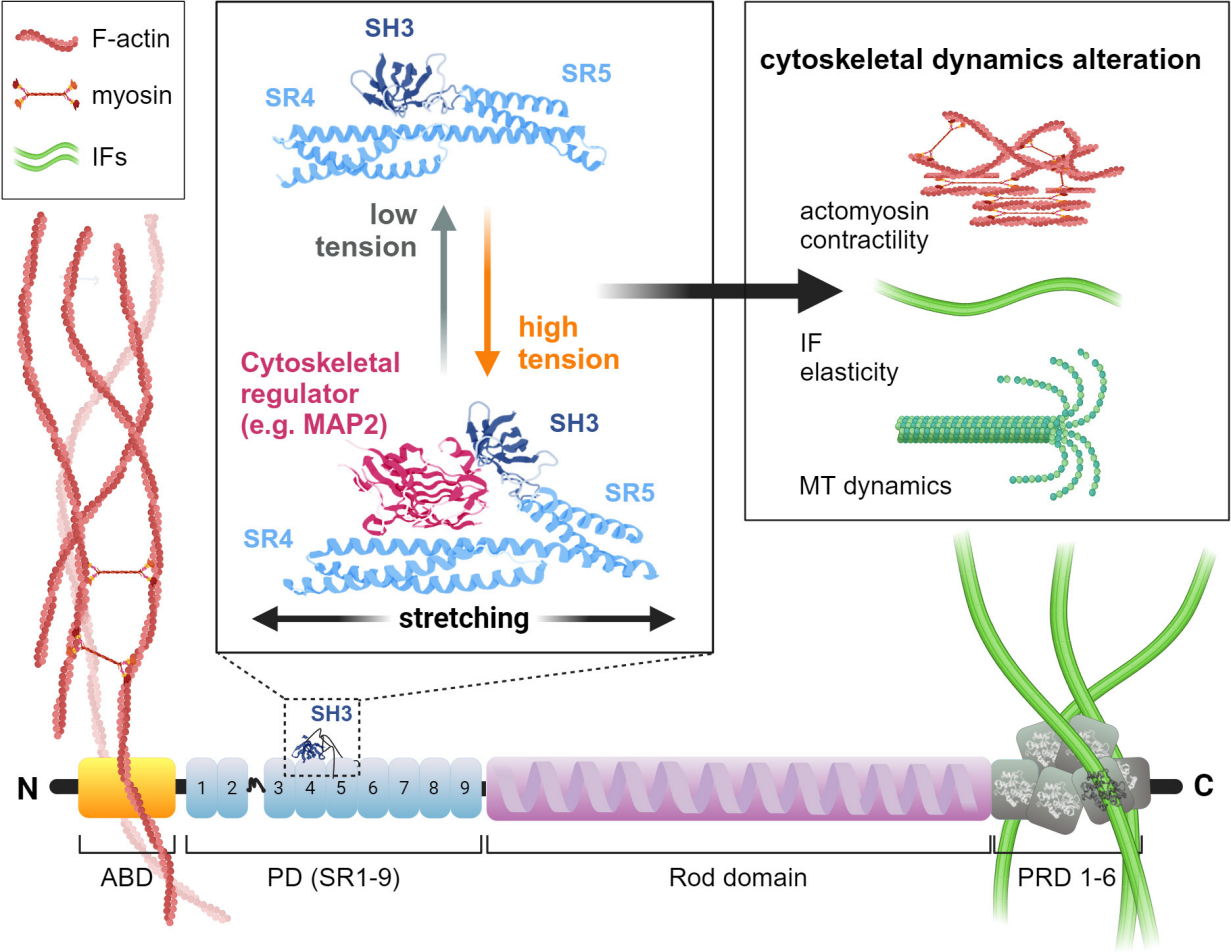
Schematic representation of plectin as a new class of cytolinker-based mechanosensors. Plectin crosslinks IFs and contractile actomyosin networks and recruits them to tension-sensitive junctions (such as FAs, HDs or DSMs; not shown, for details see §§2, 5 and 8.4). Increased tension resulting from intrinsic and extrinsic forces leads to unfolding of SR4 and SR5 within the plectin PD (middle box; PDB: 3PE0 [10]), releasing the SR4/SH3 interface from inhibitory intramolecular interactions. The unmasked SR4/SH3 interface allows the binding of ligands (such as MAP2) that regulate the dynamics of individual cytoskeletal networks (right box). Hypothetically, a similar mechanism can be envisaged for the regulation of actomyosin contractility or the flexibility/stiffness of IFs. Created in BioRender (https://BioRender.com/o88e610).

The efforts to identify mechanosensitive and mechanotransductive structural elements within the plectin molecule have primarily focused on the evolutionarily conserved PD, consisting of nine spectrin repeats (SR1–SR9), and in particular on the SH3 domain embedded within spectrin repeats [10,282]. Unlike other SH3 domains that mediate protein–protein interactions, this unique SH3 domain forms an intramolecular autoinhibitory interaction with SR4, blocking its accessibility. However, molecular dynamics simulations showed that physiological pulling forces (~500 pN) lead to the unfolding of SR4 and SR5 repeats, exposing the cryptic SH3 domain for binding [283] (figure 4). Similar results provided an analysis of the SH3 domain harboured by PD of the *Caenorhabditis elegans* plectin homologue VAB-10A [284]. Interestingly, the force required for SR unfolding is significantly decreased in the absence of the SH3 domain, suggesting that the SH3 domain itself stabilizes the folded conformation [283]. Deletion or mutagenesis of the VAB-10A SH3 domain attenuated mechanosensitive recruitment of the adaptor protein GIT-1 and induced embryonic elongation arrest [284]. *In vitro* analysis revealed that the plectin SH3 domain interacts with MAP2c, with consequences for MT stabilization and assembly [37]. As no other interactors of this domain have been identified, it is plausible that an alternative mechanism of binding to the plectin SH3 domain requires the involvement of one or more SRs [10]. This is of particular interest for putative mechanosensing since plectin SRs, including SR4–SH3–SH5, participate in the interactions with junctional proteins such as the HD integrin β4 [26,50] and BPAG2 [27] in epithelia and β-dystroglycan [46] and β-synemin in skeletal muscle [59].

In this review, we propose a simple concept where plectin crosslinks actin fibres with IFs and recruits them to supramolecular assemblies at the sites of cell attachment either to the underlying substrate (cell–ECM junctions) or to its neighbour (cell–cell junctions). Upon stretch, the SR4/SH3 interface is released from intramolecular interaction, allowing ligand binding to the unmasked SH3 domain (figure 4). Such interaction can lead to ligand sequestration and reduced availability for downstream signalling events. The scaffolding function of plectin has been well described for the RACK1, where it was shown to reduce membrane-associated PKCδ and c-Src activities [42,43]. In a similar fashion, the SH3 domain of plectin may participate in ligand activation by inducing conformational changes upon binding. It is likely, however, that the same tension-sensitive interactions with modulators of the actin dynamics may be responsible for signalling crosstalk between the IF and the actin cytoskeletal networks.

Indeed, several studies have implicated plectin in the regulation of cytoskeletal tension, with manipulations of plectin often resulting in altered actin cytoskeleton dynamics. For example, plectin deficiency was shown to increase the actomyosin contractility in various cell lines, including endothelial cells [285], fibroblasts [75], MDCK cells [22] and mouse [118] or human [119] keratinocytes. The signalling cascade affected by plectin depletion, leading to elevated actomyosin contractility has not yet been identified. However, it is likely to involve activation of the Rho-ROCK signalling pathway, as plectin depletion has been associated with increased RhoA activation [75] and MLC phosphorylation [75,119]. These findings reflect the ability of plectin to control signalling pathways that regulate actomyosin contractility, potentially providing a feedback loop to control tensional homeostasis with a defective IF networks caused by plectin mutation/ablation.

## 9. Concluding remarks and outlook

Over the last four decades, our understanding of how cytoskeletal crosslinking governs fundamental principles of epithelial mechanobiology has significantly evolved. The development of both constitutive and tissue- or cell type-specific conditional knockout and knock-in mouse models has facilitated the elucidation of the pathophysiology of plectinopathies affecting simple as well as (pseudo)stratified epithelia. The combination of high-resolution imaging and force/tension measurements (§§8.1 and 8.2) has uncovered relationship between the structural organization of cytoskeletal networks/cell junctions and the spatiotemporal distribution of actomyosin-generated tension. Furthermore, the molecular identity of key players engaged in cytoskeletal-junctional crosstalk has been identified in endogenous settings by proximity labelling- and FRET-based approaches. In light of these advances, we are only just beginning to appreciate the complexity of the intricate dance between so many molecular interactors that is required to sustain the mechanical homeostasis of epithelial sheets.

However, despite all these efforts, there are many interesting challenges for the future. It is essential to elucidate whether and how plectin (and other cytolinkers)-mediated connectivity between KFs and actomyosin networks modulates modes of contraction and local force generation. The identification of a mechanosensitive SR/SH3 plectin module has opened up another intriguing direction for future studies, with mechanosensitive ligands and downstream signalling pathways yet to be uncovered. Moreover, it seems probable, that similar principles of mechanotransduction will apply to other members of the plakin family. This new class of mechanosensors would benefit from the residency among all three cytoskeletal networks, as well as from selective targeting to mechanosensitive hubs such as cell–cell and cell–ECM junctions. Equally important is to decipher the specific functions of plectin isoforms that are (or at least some of them) recruited to different cellular structures in a tension-dependent manner. This leads to the engaging hypothesis that tension-sensitive plectin isoform patterning would allow fine-tuning of signalling hubs and provide framework for mechanical memory of the cell. In this scenario, the engagement of diverse isoform subsets, in conjunction with isoform-specific cytoskeletal components and downstream effectors would help with distinguishing between continuous and sudden mechanical cues, thereby enabling the cell to adapt to repetitive mechanical inputs (such as breathing or peristalsis). Finally, given the critical role plectin plays in cell/tissue mechanics, which is a key aspect of epithelial carcinogenesis, it is surprising that only a few studies aiming at elucidating the role of plectin in cancer have been published so far.

## Data Availability

This article has no additional data.
